# Overweight and Severe Acute Maternal Morbidity in a Low-Risk Pregnant Population in The Netherlands

**DOI:** 10.1371/journal.pone.0074494

**Published:** 2013-09-12

**Authors:** Tom Witteveen, Joost J. Zwart, Karin B. Gast, Kitty W. M. Bloemenkamp, Jos van Roosmalen

**Affiliations:** 1 Department of Obstetrics, Leiden University Medical Center, Leiden, The Netherlands; 2 Department of Obstetrics and Gynaecology, Deventer Ziekenhuis, Deventer, The Netherlands; 3 Department of Clinical Epidemiology, Leiden University Medical Center, Leiden, The Netherlands; 4 Department of Internal Medicine, Leiden University Medical Center, Leiden, The Netherlands; 5 Department of Medical Humanities, EMGO Institute for Health and Care Research, VU University Medical Centre, Amsterdam, The Netherlands; University of Arkansas for Medical Sciences, United States of America

## Abstract

**Objective:**

To investigate the association between overweight and severe acute maternal morbidity (SAMM) in a low-risk pregnant population.

**Design:**

Nationwide case-control study.

**Setting:**

The Netherlands, august 2004 to august 2006.

**Population:**

1567 cases from initially primary care and 2994 women from primary care practices as controls, out of 371 012 women delivering in the Netherlands during the study period

**Methods:**

Cases were women with SAMM obtained from a nationwide prospective study. All women in this cohort who initially had low-risk pregnancies were compared with low-risk women without SAMM to calculate odd ratios (ORs) to develop SAMM by body mass index (BMI) category. We divided body mass index in three overweight categories and calculated the ORs (95% CI) of total SAMM and per specific endpoint by logistic regression, with normal weight as reference. We adjusted for age, parity and socio-economic status.

**Main Outcome Measures:**

SAMM, defined as Intensive Care Unit (ICU)-admission, Uterine Rupture, Eclampsia or Major Obstetric Haemorrhage (MOH)

**Results:**

SAMM was reported in 1567 cases which started as low-risk pregnancies. BMI was available in 1097 (70.0%) cases and 2994 control subjects were included. Analysis showed a dose response relation for overweight (aOR, 1.3; 95% CI, 1.0-1.5), obese (aOR, 1.4; 95% CI, 1.1-1.9) and morbidly obese (aOR, 2.1; 95% CI, 1.3-3.2) women to develop SAMM compared to normal weight. Sub analysis showed the same dose response relation for ICU-admission, Uterine Rupture and Eclampsia. We found no association for MOH.

**Conclusion:**

Overweight without pre-existent co-morbidity is an important risk-indicator for developing SAMM. This risk increases with an increasing body mass index.

## Introduction

The increasing prevalence of overweight and obesity is a dramatic trend all over the world, especially in western countries. The United States has taken the lead with more than one-third of adult women being obese [1]. In the United Kingdom the prevalence of obesity at the start of pregnancy has increased from 9,9% to 16% in a 15-year period [2]. In the Netherlands the prevalence of self-reported overweight and obesity in women increased from respectively 30% and 6% in 1981 to 42% and 12% in 2004 [3]. In many other western countries similar trends are observed [4,5]. Obesity is a risk factor for chronic diseases such as cardiovascular disease and type 2 diabetes [6,7]. In obstetrics, women with overweight have a higher risk for adverse neonatal outcomes [8-12] and many studies have reported an increased risk for gestational diabetes, pre-eclampsia and caesarean delivery [13-16]. As a consequence overweight leads to increased utilisation of healthcare during pregnancy and therefore to higher costs [17-19]. We hypothesize that the increased prevalence of overweight in the Netherlands contributes to the observed increase of maternal mortality [20]. This is difficult to investigate, because numbers are low. Therefore, we used severe morbidity as an outcome measurement. In the Netherlands overall SAMM occurred in 7.1 per 1,000 births with a case fatality rate of 1 in 53 [21]. The objective of this article is to report the association between overweight and severe acute maternal morbidity (SAMM) in a low-risk pregnant population.

## Methods

### Ethics statement

The LeMMoN-study was centrally approved by the medical ethics committee of Leiden University Medical Center (P04-020; 8 March 2004). In this study only anonymous data is used and information cannot be related to individual women. In the Netherlands informed consent and ethical approval is not needed when all participant information is anonymous.

### Study design and study population

This is a nationwide case-control study investigating the association between overweight and SAMM. SAMM was defined as Intensive Care Unit (ICU)-admission, Uterine Rupture, Eclampsia, Major Obstetric Haemorrhage (MOH) or Miscellaneous (severe acute maternal morbidity according to the opinion of the treating obstetrician, which could not be included in the four other categories). Women could be included in more that one SAMM category. Cases were women with SAMM who started their pregnancy in primary care. Controls were women in primary care without SAMM. Cases were selected from the LEMMoN study, a nationwide prospective cohort study, detailed information of which was described previously [21]. In summary; all cases of severe acute maternal morbidity during pregnancy, delivery and the puerperal period were included from all 98 hospitals in the Netherlands with a maternity unit from august 1, 2004 until august 1, 2006. These hospitals consist of 8 tertiary care hospitals, 35 non-academic teaching hospitals and 55 general hospitals. We excluded cases that were referred to a secondary or tertiary care centre before 18 weeks of gestation and cases with missing data on height and weight or BMI (i.e. weight not registered before 14 weeks of gestation). The referral cut-off point of 18 weeks of gestational age to secondary care includes at least two primary care visits for risk selection.

For the controls, we collected data of all pregnant women with known BMI, who delivered between August 1, 2004 and August 1, 2005 in eight primary obstetric care practices dispersed in the Netherlands. All controls could have developed SAMM. When this happened they have been referred to secondary care and included in the LEMMoN-study case group. We conveniently choose practices that had excellent registration of BMI. By selecting women from primary obstetric care practices, we were certain to only include women without co-morbidities due to the Dutch risk selection system [22].

The Dutch risk selection system is based on risk selection in primary obstetric care where pregnant women are guided through pregnancy and referred to secondary or third specialised obstetric care when higher risk for or a present complication exists, meaning that the low-risk women used in this study do not have any pre-existent co-morbidity [22]. By including only the initially low-risk cases we corrected for possible confounding co-morbidities as the initially high-risk cases have been referred. We used measured BMI at booking because self-reported BMI have shown to be unreliable.

### Data collection

Data available for the cases included maternal characteristics (age, BMI, zip-code, parity and ethnicity) and information regarding pregnancy, delivery and the corresponding specific complication(s). This data was extracted monthly from each hospital using a standardised web based form reported by a local coordinator.

Available data for the control subjects included age, BMI, parity, zip-code, occupation, birth weight, place of delivery and mode of delivery. All used data were continuous except BMI (see statistical analysis), parity (0, 1, 2 and ≥3), ethnicity (native or immigrant, only available for cases) and socio-economic status (low, modest, high).

### Statistical analysis

We divided participants in categories according to their BMI based on the WHO-classification (underweight; BMI<18.5, normal; BMI 18.5-24.9, overweight; BMI 25.0-29.9, obesity; BMI 30.0-34.9 and morbid obesity; BMI ≥35.0). We calculated the socio-economic status score per participant by combining residence value and average income with factor analysis. This score was divided into three categories of socio-economic status (SES); low, modest and high. Residence value and average income were based on the validated residence zip-code indicator list of Statistics Netherlands (CBS) [23,24].

We examined differences in characteristics between cases and control subjects and these were tested with a chi-square test or independent t-test where appropriate. Furthermore, we investigated whether cases with known BMI differed from cases without BMI by comparing other characteristics.

We calculated crude odds ratios (OR) and their 95% confidence intervals (95% CI) of SAMM for women with a BMI<18.5, BMI≥25, (including BMI≥30 and 35), BMI≥30 (including BMI≥ 35) with normal weight (BMI 18.5-24.9) as a reference category. In a multivariable logistic regression analysis we calculated adjusted odd ratios (aOR) for age, parity and socio-economic status. In this model data were only used if age, parity and socio-economic status were known.

We additionally calculated OR (95% CI) for the different categories of SAMM (i.e. ICU-admission, Uterine Rupture, Eclampsia, MOH) except for the Miscellaneous group which was a very heterogeneous category including many different complications. Cases could be included in more than one SAMM category. In the total SAMM analyses these cases were included once. Statistical analysis was performed using SPSS statistics, version 17.0 (SPSS, Chicago, IL).

## Results

Between August 1, 2004 and August 1, 2006, 371 012 women delivered in the Netherlands according to Statistics Netherlands [23]. Out of 2552 reported SAMM cases we excluded the high-risk pregnancies at booking (n=985) and cases with missing data for BMI (n=470), 1097 cases were left for analyses. We collected data of 2994 controls with known BMI.

### Demographics

The case group included 356 (32.5%) women with ICU-admission, 71 (6.4%) with Uterine Rupture, 113 (10.2%) with Eclampsia, 704 (64.2%) with MOH and 142 cases reported as Miscellaneous (12.9%).

All compared variables showed significant differences. Cases had a higher mean BMI (24.4 kg/m^2^ versus 23.8 kg/m^2^) and had overweight more frequently. The percentage of women with a normal weight was 62.0% in the cases compared to 65.2% in the control subjects. The prevalence of overweight, obesity and morbid obesity was respectively 248 (22.6%), 70 (6.4%) and 54 (4.9%) in the cases, compared to 619 (20.6%), 200 (6.7%) and 77 (2.6%) in the control group (Table 1).

**Table 1:** Characteristics of cases and control subjects.

		**Cases N = 1097**	**Control subjects N=2994**	**P value**
Age (years)		30.9 (4.7)	30.1 (5.0)	<0.001
SES (n, %)	Low	272 (27.8)	445 (17.7)	<0.001
	Modest	476 (48.7)	1370 (54.4)	
	High	229 (23.4)	704 (27.9)	
Parity (n, %)	0	647 (59.0)	1463 (48.7)	<0.001
	1	333 (30.4)	1048 (34.9)	
	2	83 (7.6)	345 (11.5)	
	≥3	34 (3.1)	122 (4.1)	
Birth weight (gram)		3296 (834)	3490 (550)	<0.001
BMI (kg/m²)		24,4 (5.0)	23,8 (4.4)	<0.001
BMI category^1^ (n, %)	Underweight	45 (4.1)	145 (4.8)	.001
	Normal	680 (62.0)	1953 (65.2)	
	Overweight	248 (22.6)	619 (20.7)	
	Obesity	70 (6.4)	200 (6.7)	
	Morbid obesity	54 (4.9)	77 (2.6)	

Data are presented as mean (SD) or number (%)

^1^ BMI classification, see Method section

SES = Socio-Economic Status; BMI = Body Mass Index.

### Main outcome

Table 2 shows the association between BMI categories and the risk to develop SAMM. Women with overweight had an aOR of 1.3 (95% CI, 1.0-1.5) to develop SAMM compared women with a normal weight. For obese women the aOR increased to 1.4 (95% CI, 1.1-1.9) and for morbidly obese to 2.1 (95% CI, 1.3-3.2). The sub analyses for the first three inclusion groups showed a dose response increase in aOR except for morbidly obese women with Uterine Rupture. Analysis for MOH showed no significant difference (Table 2). Detailed description of the Miscellaneous group has been published previously^21^. For example, this group also includes two extreme obese women with anaesthetic complications which did not fulfil the criteria of the other four categories.

**Table 2:** Primary and secondary analysis results.

	**Underweight**		**Overweight**		**Obesity**		**Morbid Obesity**	
	OR (95% CI)	aOR* (95% CI)	OR (95% CI)	aOR* (95% CI)	OR (95% CI)	aOR>* (95% CI)	OR (95% CI)	aOR* (95% CI)
**ICU-admission** N= 356	1.2 (0.7-2.0)	1.4 (0.8-2.5)	1.2 (1.0-1.6)	1.4 (1.1-1.9)	1.6 (1.1-2.2)	1.7 (1.1-2.6)	3.1 (1.9-4.9)	3.2 (1.8-5.9)
**Uterine rupture** N= 71	^#^	^#^	2.2 (1.4-3.5)	2.0 (1.1-3.7)	3.3 (1.8-6.1)	3.6 (1.6-7.9)	3.7 (1.4-9.8)	3.2 (0.9-11.4)
**Eclampsia** N= 113	1.4 (0.6-3.1)	1.6 (0.6-4.1)	1.3 (0.9-1.9)	1.8 (1.1-3.0)	1.4 (0.8-2.5)	2.4 (1.2-4.8)	3.4 (1.6-7.1)	6.4 (2.8-14.7)
**Major Obstetric Hemorrhage** N= 704	0.8 (0.5-1.2)	0.7 (0.4-1.2)	1.0 (0.8-1.2)	1.0 (0.8-1.3)	0.9 (0.7-1.2)	1.1 (0.8-1.5)	1.2 (0.7-1.9)	1.0 (0.5-2.0)
**SAMM Total**	0.9 (0.6-1.3)	0.9 (0.6-1.3)	1.2 (1.0-1.4)	1.3 (1.0-1.5)	1.3 (1.0-1.6)	1.4 (1.1-1.9)	2.0 (1.4-2.9)	2.1 (1.3-3.2)

^*^ Adjusted for age, parity and socio-economic status. ^#^ Not enough cases (n=3) for analysis ICU = Intensive Care Unit; CI = confidence interval; OR = odds ratio; aOR = adjusted odds ratio

## Discussion

This study shows that women with overweight had a 30% higher risk and women with obesity had a 40% higher risk to develop SAMM compared to women with a normal weight. The association between overweight and SAMM is even stronger for specific endpoints such as ICU-admission, uterine rupture and eclampsia. We found no increased risk for major obstetric haemorrhage. The increasing incidence of overweight and obesity seems to be one of the causal factors in the increasing trend in SAMM in western countries, other suggested factors being the increased age of women, the increased caesarean rate and the increased rate of multiple pregnancies through artificial reproduction techniques.

### Strengths and limitations

The incidence of SAMM is relatively low and therefore a case-control study is the most appropriate design. We were able to collect information on all cases of SAMM in the Netherlands during a two-year period and to include a large number of cases.

This study also has some limitations that need to be considered. Due to the observational and retrospective aspect of data collection, residual confounding may remain. This is inherent to the study design. The missing values for BMI in the case group could have introduced selection bias. To explore this we compared cases with known BMI to cases with missing BMI (Table 3 and S1). This analysis shows that all the initially low-risk cases are similar in seven out of nine characteristics. Cases with missing BMI had a higher incidence of immigrants, and a higher incidence of women with low socio-economic status than the cases with known BMI. Both factors are associated with higher BMI and thus due to this exclusion our results are likely to be an underestimation.

**Table 3:** Comparison of low-risk cases to excluded cases without BMI (For all compared characteristics: see Table S1).

		**BMI**	**BMI missing**	
		**N=1097**	**N=470**	**P-value**
SES (n, %)	Low	272 (27.8)	153 (36.6)	<0.01
Missing = 171	Modest	476 (48.7)	175 (41.9)	
	High	229 (23.4)	90 (21.5)	
Ethnicity (n, %)	Native	870 (79.5)	333 (71.3)	<0.001
Missing = 5	Immigrant	225 (20.5)	134 (28.7)	

BMI=Body Mass Index; SES=Socio-Economic Status.

Data are presented as number (%)

The exclusion of confounding of co-morbidities relies on the quality of the Dutch risk selection system in which women with co-morbidities should be referred to secondary care. Appropriate application of these guidelines has been confirmed by previous studies. For example, Zwart et al. showed that women delivering under the care of a Dutch primary care giver have a lower risk to develop SAMM (RR 0.1 95% CI: 0.1-0.2) [21]. Assuming that complications before 18 weeks are followed by referral to secondary care, the calculated risks are primarily the consequence of overweight without overt consequences of their overweight. The women selected initially as low-risk probably had underlying pathology, such as co-morbidities that were clinically not (yet) present. Referral indication primarily based on high BMI in the absence of any other known pathology was not advised in the national guideline used during the study period.

To collect controls, we conveniently selected primary care practices that had an excellent registration of BMI. At the moment of selection, we were not aware of the actual BMI values in the practices. Furthermore, rates of BMI categories corresponded well with national incidence figures from Statistics Netherlands (CBS) during the study period (National: BMI≥25: 31.7% and BMI≥30: 9.1%; Control population: BMI≥25: 30.0% and BMI≥30: 9.3%) [21,23].

Ethnicity information was not available for the control group and therefore adjustment was not possible. Previous studies showed higher risks for immigrant women to develop adverse pregnancy outcomes [21,27-32]. Due to this limitation there could be residual confounding in our primary results caused by ethnicity. Our results also show wide confidence intervals for specific conditions of SAMM due to low numbers: for example, the OR of morbidly obese women with uterine rupture (n=5). This rare situation with non-significant OR could still be considered clinically relevant.

### Interpretation and comparison

Maternal overweight could have a harmful effect in different phases of pregnancy and the postpartum period; during risk-assessment, pregnancy and labour monitoring and delivery. In the antenatal phase ultrasonography on overweight women has shown to go with difficulties visualising fetal structures between 18 and 24 weeks and therefore assessing potential risks [33]. Also, the measurement of blood pressure has shown to be less accurate in women with overweight and this may lead to delayed detection of (pre-) eclampsia. If there is a potential risk detected at home, then the difficulty of transport arises in the extremely obese. Elevated risks during the perinatal phase can be the consequence of a delay in induction, a longer duration of labor, higher incidence of caesarean section and difficulty with anaesthesiology. For example, Pevzner et al. [34] showed an almost twice the amount of predelivery oxytocin units was needed in labour induction (BMI<30, 2.6 units; BMI>40, 5.0 units; p<0.001). They also showed a more than four hour (p<0.001) longer duration of labor for BMI>40(27.0 hour) compared to women with a BMI<30(22.7 hour) [34]. The elevated risk for emergency and elective caesarean section in overweight women is supported by many large studies [14,35,36]. This goes along with peri-operative problems such as the placement of an epidural catheter or tracheal tube in obese patients [37]. For example, a six-year review of failed intubation in 36 obstetric patients out of 8970 (incidence 1:249) general anaesthetics, showed an average BMI of 33 in the UK [38]. Besides the procedural difficulties there are also risks for overweight women in the operation room. The physiological differences compared with a normal weight non-pregnant woman further complicate the whole process of delivery and anaesthesiology [39]. Adding to this, there are also increased infectious risks during and after delivery. Sebire et al. showed significant risks for overweight women to develop genital tract, urinary tract and wound infection compared to normal weight (BMI 20-<25) women [14]. The higher risk for infectious morbidities and the decreased performance in general can explain the increased risk for overweight women to be admitted to an intensive care unit. The high incidence of previous caesarean section in overweight women also explains the elevated risk for uterine rupture [40,41]. This is the reason that in this study caesarean section was not considered as a potential confounder. Also no adjustment was performed for birth weight because studies show a significant relation with overweight [14,25-28].

Interestingly, no significantly increased risk was observed for major obstetric haemorrhage as endpoint in this study. Large previous studies do not support this finding, although we mention that these studies did not study low-risk populations. Cedergren et al. [28] showed aOR’s of 1.19 (BMI 29.1-35; 95% CI, 1.15-1.23), 1.36 (BMI 35.1-40; 95% CI, 1.25-1.48) and 1.70 (BMI>40; 95% CI 1.45-1.98) among vaginally delivered women to develop major postpartum haemorrhage [28]. Sebire et al. used a cut-off value of >1000 ml and found aOR’s of 1.17 (BMI 25-30; 99% CI, 1.07-1.27) and 1.44 (BMI>30; 99% CI, 1.30-1.60) [14]. As blood loss is underestimated [42,43] and blood transfusion depends on local management these OR’s are difficult to compare. In the LEMMoN study only cases needing transfusion of at least 4 units of packed red blood cells were included and we did not find a relation with overweight.

As the prevalence of overweight increases rapidly, the incidence of SAMM and probably maternal mortality will likely increase in the future. In our opinion morbidly obese women (BMI≥35) should be included in the national guidelines as “official risk factor” as reason for referral. For obese (BMI≥30) women, we advise midwives or obstetricians to thoroughly evaluate these patients with an individual perspective. When other SAMM risk factors such as a previous caesarean section or a previous severe preeclampsia are present these patients should also be referred. Only if the obesity epidemic will be put to a hold the consequences might be attenuated. As weight loss during pregnancy is contraindicated, SAMM and life threatening complications can only be avoided by preconceptional counselling to stimulate weight loss and weight monitoring during pregnancy of overweight women.

## Conclusion

This study shows that maternal overweight without pre-existent co-morbidities is an important risk-factor for SAMM in a Dutch low-risk population. Obese and morbidly obese pregnant women should be regarded as high risk pregnant women also in the absence of any overt co-morbidity.

## Supporting Information

Table S1 **Characteristics of included and excluded low-risk cases.**
(DOC)
