# Supplementary material for: Overweight and Severe Acute Maternal Morbidity in a Low-Risk Pregnant Population in The Netherlands
Source: PLoS One. 2013 Sep 12;8(9):e74494. doi: 10.1371/journal.pone.0074494 (PMC3772123; doi:10.1371/journal.pone.0074494)
Supplement: Table S1 — Characteristics of included and excluded low-risk cases. (DOC) [file pone.0074494.s001.doc]

**Table S1. Characteristics of included and excluded low-risk cases**

| **Total number cases**  **N = 1567** |  |  |  |  |
| --- | --- | --- | --- | --- |
|  |  | **BMI**  **N=1097** | **BMI missing**  **N=470** | **P-value** |
| Age (years) |  | 30.9 (4.7) | 30.5 (5.2) | .177 |
|  | Missing = 2 |  |  |  |
| SES (n, %) | Low | 272 (27.8) | 153 (36.6) | <0.01 |
|  | Modest | 476 (48.7) | 175 (41.9) |  |
|  | High | 229 (23.4) | 90 (21.5) |  |
|  | Missing = 171 |  |  |  |
| Parity (n, %) | 0 | 648 (59.1) | 249 (53.2) | .118 |
|  | 1 | 332 (30.3) | 154 (32.9) |  |
|  | 2 | 83 (7.6) | 44 (9.4) |  |
|  | ≥3 | 34 (3.1) | 21 (4.5) |  |
|  | Missing = 2 |  |  |  |
| Etnicity (n, %) | Native | 870 (79.5) | 333 (71.3) | <0.001 |
|  | Immigrant | 225 (20.5) | 134 (28.7) |  |
|  | Missing = 5 |  |  |  |
| Hospital admission (days) |  | 8,2 (8.9) | 7.6 (7.0) | .148 |
|  | Missing = 55 |  |  |  |
| Birth weight (gram) |  | 3179 (976) | 3155 (1044) | .668 |
|  | Missing = 92 |  |  |  |
| Max. Diastolic blood pressure (mmHg/min) |  | 87 (18) | 88 (19) | .724 |
|  | Missing = 172 |  |  |  |
| Blood loss (ml) |  | 2316 (2135) | 2268 (2071) | .698 |
|  | Missing = 142 |  |  |  |
| Gestational age (weeks) |  | 35,8 (6,2) | 35,8 (6,3) | .917 |
|  | Missing = 79 |  |  |  |

BMI=Body Mass Index; SES=Socio-Economic Status.

Data are presented as mean (SD) or number (%)
